# Mesh-related complications and recurrence after ventral mesh rectopexy with synthetic versus biologic mesh: a systematic review and meta-analysis

**DOI:** 10.1007/s10151-021-02534-4

**Published:** 2021-11-23

**Authors:** E. M. van der Schans, M. A. Boom, M. El Moumni, P. M. Verheijen, I. A. M. J. Broeders, E. C. J. Consten

**Affiliations:** 1 Department of Surgery, Meander Medical Center, Maatweg 3, 3813 TZ Amersfoort, The Netherlands; 2 Faculty of Electrical Engineering, Mathematics and Computer Science, Institute of Technical Medicine, Twente University, Enschede, The Netherlands; 3 Department of Surgery, University of Groningen, University Medical Center Groningen, Groningen, The Netherlands

**Keywords:** Rectal prolapse, Rectopexy, Erosion, Recurrence, Systematic review, Meta-analysis

## Abstract

**Background:**

Ventral mesh rectopexy (VMR) is a widely accepted surgical treatment for rectal prolapse. Both synthetic and biologic mesh are used. No consensus exists on the preferred type of mesh material. The aim of this systematic review and meta-analysis was to establish an overview of the current literature on mesh-related complications and recurrence after VMR with synthetic or biologic mesh to aid evidence-based decision making in preferred mesh material.

**Methods:**

A systematic search of the electronic databases of PubMed, Embase and Cochrane was performed (from inception until September 2020). Studies evaluating patients who underwent VMR with synthetic or biologic mesh were eligible. The MINORS score was used for quality assessment.

**Results:**

Thirty-two studies were eligible after qualitative assessment. Eleven studies reported on mesh-related complications including 4001 patients treated with synthetic mesh and 762 treated with biologic mesh. The incidence of mesh-related complications ranged between 0 and 2.4% after synthetic versus 0–0.7% after biologic VMR. Synthetic mesh studies showed a pooled incidence of mesh-related complications of 1.0% (95% CI 0.5–1.7). Data of biologic mesh studies could not be pooled. Twenty-nine studies reported on the risk of recurrence in 2371 synthetic mesh patients and 602 biologic mesh patients. The risk of recurrence varied between 1.1 and 18.8% for synthetic VMR versus 0–15.4% for biologic VMR. Cumulative incidence of recurrence was found to be 6.1% (95% CI 4.3–8.1) and 5.8% (95% CI 2.9–9.6), respectively. The clinical and statistical heterogeneity was high.

**Conclusions:**

No definitive conclusions on preferred mesh type can be made due to the quality of the included studies with high heterogeneity amongst them.

## Introduction

Since its introduction in the early ‘00, ventral mesh rectopexy (VMR) has received wide acceptance among colorectal surgeons as a minimally invasive, safe and effective procedure to treat rectal prolapse. During this operation a surgical mesh is sutured to the ventral aspect of the rectum and attached to the sacral promontory [1]. Over the past two decades numerous studies have shown promising results regarding postoperative complications, recurrence and functional outcome compared to other rectal prolapse treatments [2–4]. These findings have promoted VMR to being one of the most widely practiced surgical treatments for rectal prolapse across the world [5, 6].

Although there is consensus about the indication for VMR i.e. external rectal prolapse (ERP) or symptomatic high-grade internal rectal prolapse (IRP) consensus on the type of mesh material to be preferred is less clear [7]. When VMR was introduced at the beginning of this century, synthetic mesh was the standard material with polypropylene and polyester grafts being the most widely used materials. However, since the United States Food and Drug Administration (FDA) report in 2011 on transvaginal mesh and the subsequent ban on the use of transvaginal mesh as of April 2019, concerns have also risen about the safety of these materials used in abdominal prolapse surgery [8, 9]. The reasons for concern are the risk of mesh exposure and infection, fistula formation and chronic pelvic pain/dyspareunia. Although these complications seem rare, with incidences being described up to 3.6%, their severity and long‐term sequelae can have such a significant impact on the quality of life, that there is reason to look for alternatives such as biologic mesh [10–12]. With biologic mesh, the risk of mesh-related complications is assumed to be lower due to the process of degradation and eventual resorption of the graft and regeneration of host tissue. On the other hand, this very degradation itself and regeneration is suspected to give more recurrences in the long term. Not insignificant is the fact that the current costs of biologic grafts are a 10–20-fold higher when compared to synthetic implants.

Two systematic reviews have been published since the introduction of biologic grafts in VMR [9, 13]. Both of them showed no clear difference in favor of either biologic or synthetic mesh regarding mesh exposure and/or recurrence. Since then, new cohort studies on biologic mesh VMR have been published.

The aim of this systematic review and meta-analysis was to establish an overview of the currently available literature regarding minimal-invasive VMR in order to determine the incidence of mesh-related complications and recurrences after utilizing synthetic versus biologic mesh. In doing so, we thus looked for evidence supporting the preference of biologic over synthetic grafts, as a justification to the higher costs that come with biologic materials.

## Materials and methods

This systematic review was conducted and reported following the Preferred Reporting Items for Systematic Reviews and Meta-Analyses (PRISMA) guidelines [14]. Search strategies, eligibility criteria, the used critical appraisal tool, and outcomes of interest were pre-specified. We did not register a review protocol in advance.

### Eligibility criteria

To obtain an overview of mesh-related complications and recurrence after synthetic and biologic VMR, studies were considered eligible if they: (1) included patients treated for external rectal prolapse (ERP) or symptomatic high-grade internal rectal prolapse (IRP); (2) used the minimal-invasive technique based on the procedure described by D’Hoore and Pfenninckx [1]; (3) included at least 10 patients; (4) accounted for mesh-related complications and/or recurrence as an outcome variable. Studies were excluded when they: (1) studied outcome after VMR combined with sacrocolpopexy (i.e. sacrocolpo-rectopexy) for the treatment of multicompartment pelvic organ prolapse; (2) were written in another language than English; (3) did not represent an original article; (4) reported on (parts of) the same study population. When the latter was apparent, the study with the smallest number of patients was excluded.

### Search strategy

The electronic databases of Pubmed, Embase and Cochrane were searched to identify relevant studies from inception until September 2020. The article search was conducted with the following terms: (ventral OR anterior) AND (rectopex*). After excluding duplicate reports, two researchers independently screened all studies on title and abstract, and subsequent full text reading of the selected studies was performed (EMS and MAB). Finally, the reference lists of the eligible studies were screened for possibly relevant articles. Disagreement amongst the authors on the quality or relevancy of articles was resolved through discussion until consensus was reached.

### Data collection

The outcome parameters of interest were mesh-related complications and/or recurrence. Mesh-related complications were defined as the symptomatic or asymptomatic presence of mesh exposure, mesh infection, fistula formation and/or spondylodiscitis. Recurrence was defined as a recurrent ERP or IRP on physical examination, and/or additional imaging during follow-up, or a re-intervention for recurrent rectal prolapse. To consider a diagnosis of recurrent IRP, we required it to be associated with functional complaints (*i.e.* obstructed defecation or fecal incontinence). Conversely, mucosal prolapse following rectopexy was not considered to be a true recurrence.

When the type of mesh was not specified, the corresponding authors were contacted and asked for additional data on the mesh used. Authors were also contacted in case a mix of VMR patients and sacrocolpo-rectopexy patients were included without describing the groups separately. Finally, authors of overlapping cohorts were contacted with the request to differentiate the group of patients and their outcomes that were reported on more than once.

Data of eligible studies were collected in a pre-specified form. The following data were collected: author, year, study design, number of patients, sex, age, number of interventions for recurrent prolapse, indication for surgery (ERP or IRP), type of mesh, type of material used for fixation to the rectum, months of follow-up, the incidence of mesh-related complication and recurrence.

### Risk of bias

Two authors (EMS and MAB) independently assessed the methodological quality of each article using the methodological index for non-randomized studies (MINORS) quality score. A maximum score of 16 could be achieved for noncomparative studies, and the maximum score for comparative studies was 24 [15]. Item 4 of the MINORS score was scored separately for each outcome variable of interest (*i.e.* recurrence and mesh complications). The score of each article was noted, expressed as a total sum as well as a percentage of the maximum possible score. Studies with a score > 35% of the maximum score were considered for further meta-analysis.

### Statistical analysis

A fully open-source, cross-platform software kit for advanced meta-analyses “openMeta[Analyst]™” version 2.2 was employed. Meta-analysis was performed of the incidences of both the mesh-related complications and the recurrences. The number of events from each study were extracted and combined in a random-effects model only when more than three studies reported on the same outcome variable (i.e. the incidence of mesh complications or recurrence). Because these outcome variables were often reported as amounting zero events, an arcsine transformation was used to calculate the overall incidence. After back transformation, the pooled incidence and their 95% confidence interval (CI) were reported and plotted using forest plots. A *p* value less than 0.05 was considered statistically significant. Statistical heterogeneity was assessed using the *I*^2^-index. Subgroup analyses were performed to explore heterogeneity among the results of studies.

## Results

**Fig. 1:**
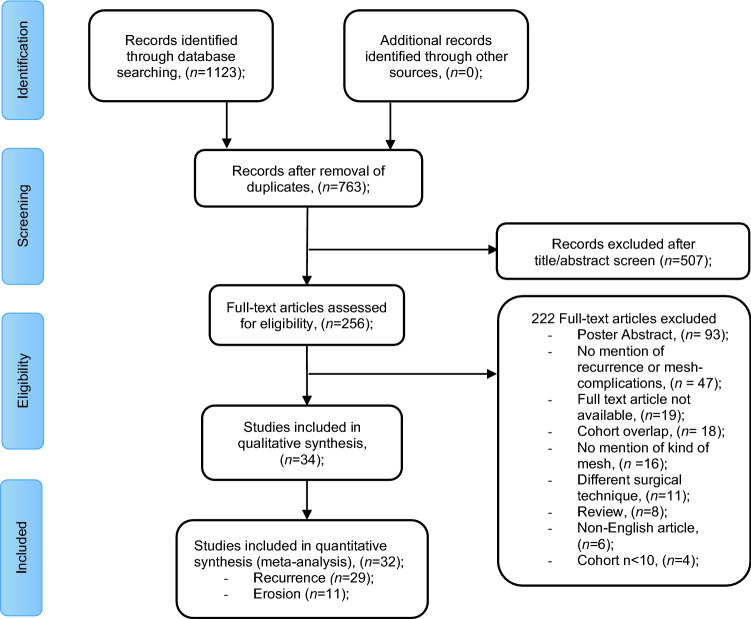
Preferred Reporting Items for Systematic Reviews and Meta-Analyses study flow diagram showing selection of studies

The database searches identified 1128 records. After exclusion of duplicates, 763 articles remained. Assessment of title and abstract led to the selection of 256 articles for full text evaluation. Of these studies, 62 studies met the inclusion criteria. Overlap among cohorts resulted in the exclusion of an additional 18 studies. Corresponding authors of 17 other articles were contacted with a request to clarify their study data of which 7 authors responded. The corresponding papers of the non-responding authors were excluded. This finally resulted in 34 articles included in this systematic review (Fig. 1).

### Quality assessment—Table 1

**Table 1:** Quality assessment using the MINORS score

Mesh	Study	MINORS criteria									Additional criteria for comparative studies				Total	
		1	2	3	4.M	4.R	5	6	7	8	9	10	11	12	*M* (% of total)	*R* (% of total)
Biologic	Brunner [20]	2	2	1	1	1	0	1	0	0	NA	NA	NA	NA	7 (44%)	7 (44%)
	Albayati [36]	2	2	1	NA	1	0	2	1	0	NA	NA	NA	NA	NA	9 (56%)
	Franceschilli [43]	2	2	2	▲	2	1	2	2	0	NA	NA	NA	NA	NA	13 (81%)
	Mehmood [42]	2	2	2	NA	2	0	2	2	0	NA	NA	NA	NA	NA	12 (75%)
	Wahed [37]	1	2	2	▲	1	0	1	2	0	NA	NA	NA	NA	NA	9 (56%)
Synthetic	Farag [38]	2	2	2	NA	2	1	1	0	0	NA	NA	NA	NA	NA	10 (63%)
	Laitakari [44]	2	2	2	NA	1	2	2	1	0	NA	NA	NA	NA	NA	12 (75%)
	Postillon [16]	1	2	1	0	2	0	1	0	0	NA	NA	NA	NA	5 (31%)	7 (44%)
	Tsunoda [21]	2	2	1	0	2	0	1	1	0	NA	NA	NA	NA	7 (44%)	9 (56%)
	Hidaka [3]	2	2	2	1	2	0	2	1	1	NA	NA	NA	NA	11 (69%)	12 (75%)
	Tejedor [25]	2	2	1	1	NA	0	1	0	1	NA	NA	NA	NA	8 (50%)	NA
	Ahmad [22]	2	1	2	0	0	0	2	0	0	NA	NA	NA	NA	7 (44%)	7 (44%)
	Chandra [31]	1	1	1	NA	1	0	2	1	0	NA	NA	NA	NA	NA	7 (44%)
	Mäkelä-Kaik. [23]	2	2	1	1	NA	0	1	1	0	NA	NA	NA	NA	8 (50%)	NA
	Emile [45]	2	2	2	NA	2	0	2	2	2	NA	NA	NA	NA	NA	14 (88%)
	van Iersel [24]	2	2	1	1	1	0	1	1	0	NA	NA	NA	NA	8 (50%)	8 (50%)
	Inaba [17]	1	1	1	0	0	0	1	0	0	NA	NA	NA	NA	4 (25%)	4 (25%)
	Luglio [34]	2	1	1	NA	2	0	2	0	0	NA	NA	NA	NA	NA	8 (50%)
	Silveira [32]	1	1	2	NA	1	0	2	0	0	NA	NA	NA	NA	NA	7 (44%)
	Horisberger [33]	2	1	0	NA	0	0	1	2	0	NA	NA	NA	NA	NA	6 (38%)
	Consten [10]	2	2	1	1	1	0	1	1	0	NA	NA	NA	NA	8 (50%)	8 (50%)
	Gosselink [39]	2	2	1	NA	2	0	2	1	0	NA	NA	NA	NA	NA	10 (63%)
	Owais [28]	1	1	2	▲	0	0	2	1	0	NA	NA	NA	NA	NA	7 (44%)
	Badrek-Am. [29]	2	1	2	▲	0	0	1	1	0	NA	NA	NA	NA	NA	7 (44%)
	Maggiori [40]	2	2	2	NA	1	0	2	1	0	NA	NA	NA	NA	NA	10 (63%)
	Tranchart [18]	1	2	0	1	NA	0	0	0	0	NA	NA	NA	NA	4 (25%)	NA
	Faucheron [26]	1	2	2	2	2	0	2	2	0	NA	NA	NA	NA	11 (69%)	11 (69%)
	Wijffels [30]	2	1	2	▲	1	0	1	1	0	NA	NA	NA	NA	NA	8 (50%)
	Boons [35]	2	2	2	▲	2	0	1	1	0	NA	NA	NA	NA	NA	10 (63%)
	Collinson [41]	2	2	2	▲	1	0	1	2	0	NA	NA	NA	NA	NA	10 (63%)
	D'Hoore [1]	2	2	2	NA	1	0	2	1	0	NA	NA	NA	NA	NA	10 (63%)
Mix	Gleditsch [19]	2	1	1	0	2	0	1	0	0	NA	NA	NA	NA	5 (31%)	7 (44%)
	Fu [27]	2	2	1	2	2	0	2	2	0	1	1	0	1	14 (58%)	14 (58%)
	Evans [12]	2	2	1	2	NA	0	1	0	0	1	1	0	2	12 (50%)	NA

MINORS criteria: 1. Clearly stated aim of study; 2. Inclusion of consecutive patients; 3. Prospective data collection; 4. Endpoint appropriate to study aim; 5. Unbiased assessment of endpoint; 6. Follow-up appropriate for study aim; 7. Loss to follow-up < 5%; 8. Prospective calculation of study size; 9. Adequate control group; 10. Contemporary groups; 11. Baseline equivalence of groups; 12. Adequate statistical analysis. The items are scored 0 (not reported), 1 (reported but inadequate) or 2 (reported and adequate). ▲ Mesh-related complications were not scored as study results were also included in Evans et al. [12]

*MINORS* methodological index for non-randomized studies, *M* mesh-related complications, *NA* not applicable, *R* recurrence

All included studies were assessed with the MINORS tool. As a result, a score of 36–50% was qualified as low quality; 51–75% as medium quality and 76–100% as high quality.

Fifteen studies reported on mesh-related complications, including four studies [16–19] that were not considered for further analysis due to failing the quality assessment. The remaining 11 studies included eight studies of low quality [10, 12, 20–25] and three studies of medium quality [3, 26, 27] (Table 1).

Thirty studies reported on recurrence, including one study that was not considered for further analysis after quality assessment [17]. The remaining 29 studies included 13 studies of low quality [10, 16, 19, 20, 22, 24, 28–34], 11 studies of medium quality [1, 21, 26, 27, 35–41], and five studies as high quality [3, 42–45] (Table 1).

### Study characteristics—Table 2

**Table 2:** Baseline characteristics of the included studies

Mesh	Study	Design	*N* ^a^	Female	Age (years)^b^	ERP	Hx RP surgery	Mesh type	FU (mo)^b^	Mesh compl	Recurrence
Biologic	Brunner [20]	R	123	95%	63 (23–92)	14%	20%	PM (84%); BD (16%)	29 (6–58)	0%	3.3%
	Albayati [36]	R	51	100%	57 ± 3	18%	8%	BD (100)	23 ± 1	NR	5.9%
	Franceschilli [43]	P	100	100%	63 ± 13	0%	5%	PM	20 (6–54)	▲	14.0%
	Mehmood [42]	RCT	51	94%	59 (25–89)	100%	35%	BD	12	NR	0%
	Wahed [37]	P	65	95%	62 (31–89)	42%	26%	PM	12 (1–29)	▲	4.6%
Synthetic	Farag [38]	P	60	100%	48 ± 9	100%	0%	PP	12	NR	3.3%
	Laitakari [44]	RCT	26	100%	63 ± 11	20%	0%	PE	60	NR	15.4%
	Postillon [16]	R	76	90%	62 (16–90)	100%	13%	PP	31 (2–92)	▽	13.2%
	Tsunoda [21]	R	50	90%	80 (40–94)	100%	19%	PP	49 (6–92)	0%	10.0%
	Hidaka [3]	RCT	38	92%	57 (IQR 42–73)	100%	0%	PP	73 (IQR 65–82)	0%	7.9%
	Tejedor [25]	R	199^c^	92%	66 (IQR 27)	NS	NR	PP	5 (IQR 10)	0%	NR
	Ahmad [22]	P	58	100%	63 ± 15	36%	14%	PVDF-PP	35 ± 14	0%	5.2%
	Chandra [31]	R	25	40%	38 (14–68)	100%	NR	PP	34 (6–82)	NR	4.0%
	Mäkelä-Kaik. [23]	R	508	95%	64 ± 16	56%	7%	PE (84%); PP (10%); other^d^ (3%)	44 (1–105)	1.4%	NR
	Emile [45]	RCT	25	68%	37 ± 7	100%	0%	PP	18 ± 5	NR	8.0%
	van Iersel [24]	R	258	96%	60 ± 14	19%	4%	PP	24 ± 22	0%	4.7%
	Luglio [34]	R	20	100%	Median 68	100%	NR	PP	12	NR	2.4%
	Silveira [32]	R	71	100%	58 ± 13	34%	28%	PE	18	NR	16.9%
	Horisberger [33]	NR	27	100%	60 (24–78)	0%	NR	PP	22 (2–39)	NR	3.7%
	Consten [10]	R	919	95%	55.8	26%	NR	PP	34 (0.4–144)	0.9%	7.4%
	Gosselink [39]	R	91	95%	61 (18–91)	45%	0%	PP	12	NR	4.4%
	Owais [28]	P	68	0%	35 (IQR 18–51)	26%	26%	PP^e^ (46%); PE (32%); PP (21%), other (1%)	42 (IQR 26–61)	▲	2.9%
	Badrek-Am. [29]	P	48	79%	43 (18–80)	23%	19%	PP	33 (1–186)	▲	12.5%
	Maggiori [40]	P	33	88%	64 ± 14	61%	NR	PP	42 ± 7	NR	6,1%
	Faucheron [26]	P	175	90%	58 (16–94)	100%	NR	PE	74 (24–181)	0.6%	1.1%
	Wijffels [30]	P	80	98%	84 (80–97)	100%	41%	PP	23 (2–82)	▲	2.5%
	Boons [35]	P	65	92%	72 (16–93)	100%	NR	PP	19 (3–41)	▲	1.5%
	Collinson [41]	P	75	92%	58 (25–88)	0%	NR	PP	12 (3–48)	▲	5.3%
	D'Hoore [1]	P	42	90%	50 (22–88)	100%	12%	PP	61 (29–98)	NR	4.8%
Mix	Gleditsch [19]	R	22	NR	NR	100%	0%	S: PP^e^ (41%); B: PM (59%)	Median 29	▽	S: 11.1% B: 15.4%
	Fu [27]	R	231	100%	64 (IQR 51–73)	49%	17%	S: PP (15%); B: PM (< 1%); BD (86%)	47 (IQR 29–63)	0%	S: 18.8% B: 6.0%
	Evans [12]	R	2203	93%	56 (15–82)	28%	NS	S: PP (60%); PP^d^ (7%); PE (13%); B: PM (14%); BD (6%)	36 (0–162)	S: 2.4% B: 0.7%	NR

*BD*  biodesign, *B* biologic mesh, *IQR* inter quartile range, *N* number, *NR* not reported, *P* prospective, *PE* polyester, *PM* permacol, *PP* polypropylene, *PVDF* polyvinylidene fluoride, *R* retrospective, *RCT* randomized controlled trial, *RP* rectal prolapse, *S* synthetic mesh

^a^Number of minimal-invasive VMRs

^b^Mean ± SD or median (range) or IQR

^c^A part of this cohort was also used in Evans [12], only patients not evaluated by Evans are depicted and considered for analysis

^d^Not specified

^e^With a component of another synthetic material; ▲ Results already evaluated in Evans [12]; ▽ results not considered for analyses because of unclear definition by the original study

The characteristics of the studies included for analysis are shown in Table 2. Publication dates varied from 2004 to 2020. Fifteen studies had a retrospective design [10, 12, 16, 19–21, 23–25, 27, 31, 32, 34, 36, 39], 12 had a prospective design [1, 22, 26, 28–30, 35, 37, 38, 40, 41, 43] and four [3, 42, 44, 45] were RCT’s. In one study, the design was not specified [33]. Five studies [20, 36, 37, 42, 43] included only patients treated with biologic VMR (Permacol or Biodesign), 24 studies [1, 3, 10, 16, 21–26, 28–35, 38–41, 44, 45] presented only data on synthetic VMR (polypropylene, polyester, ultrapro, other), and three studies included both biologic and synthetic VMR patients [12, 19, 27].

Participants in the majority of studies were middle aged women. Nearly half of the studies [1, 3, 16, 19, 21, 26, 30, 31, 34, 35, 38, 45] with synthetic mesh included ERP patients only, compared to two out of eight for biologic mesh studies [19, 42]. However, the total percentage of ERP in synthetic and biologic VMR patients was comparable (36% vs. 35%, respectively).

### Mesh-related complications—Table 3

**Table 3:** Studies reporting on mesh-related complications after ventral mesh rectopexy

Mesh	Study	Design	*N*	Mesh type	Material for fixation to rectum	Mesh-related complications	Time to event (months)^b^	FU (months)^b^
Biologic	Brunner [20]	R	123	PM (*n* = 103) BD (*n* = 20)	Ethibond	0%	–	29 (6–58)
	Fu [27]^a^	R	199	PM (*n* = 1) BD (*n* = 198)	PDS	0%	–	47 (IQR 29–63)
	Evans [12]^a^	R	439	PM (*n* = 309) BD (*n* = 130)	NR	PM: 1.0% BD: 0%	23 (2–78)	36 (0–162)
	Tsunoda [21]	R	50	PP	Endofascial stapler	0%	–	49 (6–92)
	Hidaka [3]	RCT	38	PP	Ethibond	0%	–	73 (IQR 65–82)
	Tejedor [25]	R	199	PP	PDS	0%	–	5 (IQR 10)
	Ahmad [22]	P	58	PVDF-PP	Ethibond	0%	–	35 ± 14
	Mäkelä-Kaik. [23]	R	508	PE (*n* = 426) PP (*n* = 52) Other^c^ (*n* = 17)	Ethibond	1.4%	9 (2–29)	44 (1–105)
	van Iersel [24]	R	258	PP	Ethibond	0%	–	24 ± 22
	Fu [27]^a^	R	32	PP	PDS	0%	–	47 (IQR 29–63)
	Consten [10]	R	919	PP	Ethibond	0.9%	9 (2–48)	34 (0.4–144)
	Evans [12]^a^	R	1764	PP (*n* = 1325) PP^d^ (*n* = 160) PE (*n* = 279)	NR	PP: 1.7% PP^d^: 0.6% PE: 6.5%	23 (2–78)	36 (0–162)
	Faucheron [26]	P	175	PE	Titanium staples	0.6%	9	74 (24–181)

*BD* biodesign, *IQR* inter quartile range, *N* number, *P* prospective, *PE* polyester, *PM* permacol, *PP* polypropylene, *PVDF* polyvinylidene fluoride, *R* retrospective, *RCT* randomized controlled trial

^a^Results of studies evaluating a mix of synthetic and biologic mesh are split up

^b^Mean ± SD or median (range) or IQR

^c^Not specified

^d^With a component of another synthetic material

The included studies describe a total of 4763 patients eligible for analysis of mesh-related complications. 4001 patients (84%) were treated with synthetic mesh and 762 patients (16%) were treated with a biologic implant. The type of material used for distal mesh fixation is shown in Table 3. Median or mean follow-up ranged between 5 and 74 months for synthetic mesh studies and between 29 and 47 months for biologic mesh studies. Ten (91%) studies [3, 10, 12, 20–24, 26, 27] had a median or mean follow-up of 2 years or longer. The time to event (mesh exposure) ranged from 2 to 78 months.

In the synthetic mesh studies, 58 mesh-related complications were reported in total with proportions ranging between 0 and 2.4%. The most widely used synthetic mesh was a complete polypropylene graft (*N* = 2873). A polyester graft was implanted in 880 patients.

In the biologic mesh studies, three mesh-related complications were found, all of them in one of the three studies (0.7%) [12]. In the other 2 [20, 27] studies no mesh-related complications were reported. Permacol was used in 410 patients overall and Biodesign in 348 patients. The three mesh-related complications all occurred in patients treated with a Permacol mesh.

**Fig. 2:**
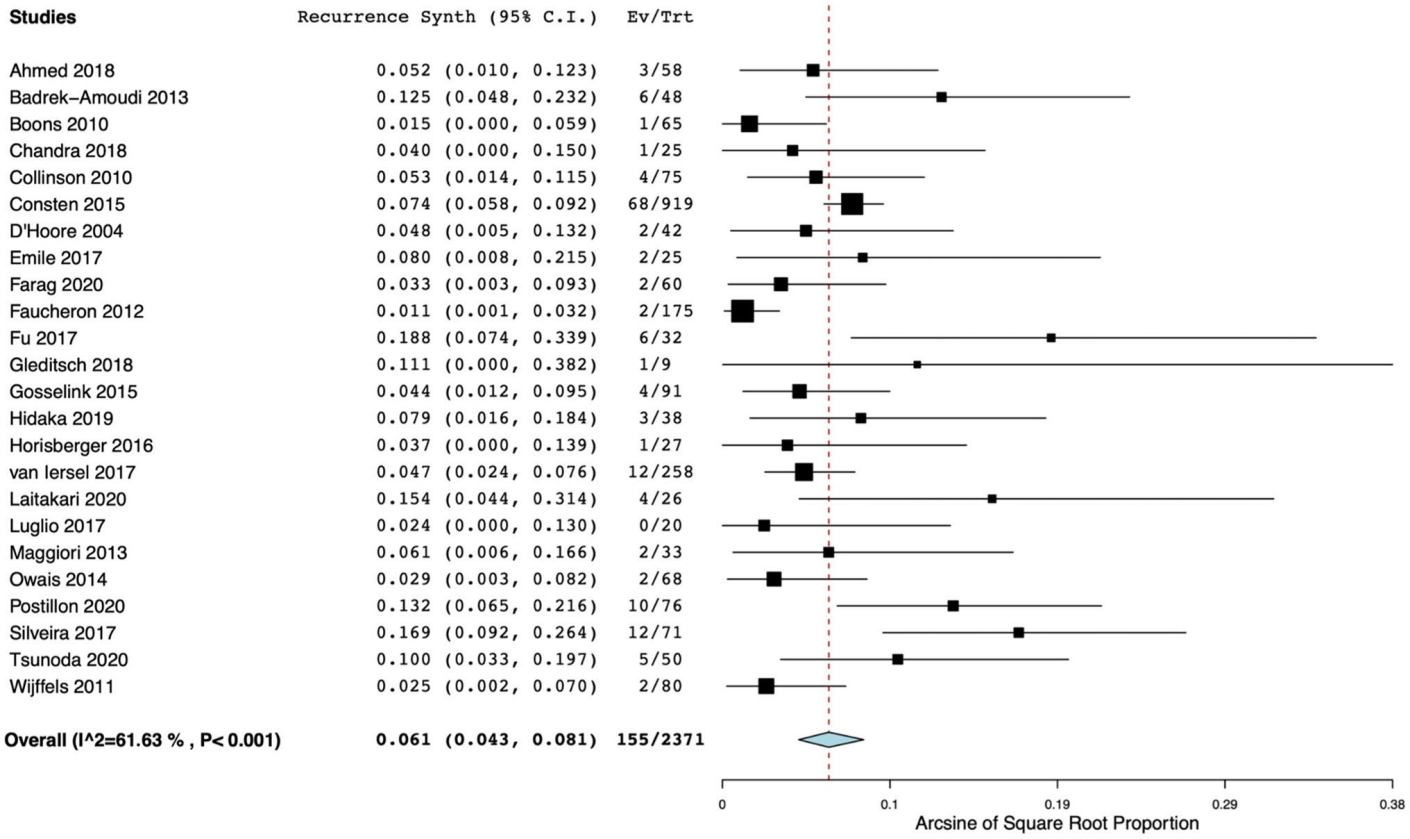
Forest plot of rates mesh-related complications after ventral mesh rectopexy with synthetic mesh. Horizontal axis for Arcsine of Square proportion; Vertical axis for included studies in meta-analyses. *Synth* synthetic, *C.I.* confidence interval, *Ev* events, *Trt* total treated with synthetic mesh

Data from synthetic mesh studies were pooled and showed a weighted mean of 1.0% (95% CI 0.5–1.7). There was strong evidence of statistical heterogeneity among these studies (*I*^2^ = 58%) (Fig. 2)). Subgroup analysis was not possible because too few studies remained (≤ 3) in the predefined subgroups. Due to the limited number of studies in the biologic mesh group reporting on mesh complications, data could not be pooled.

### Recurrence—Table 4

**Table 4:** Studies reporting on recurrence after ventral mesh rectopexy

Mesh	Study	Design	*N*	Mesh type	ERP (%)	Previous RP surgery	Recurrence rate (%)	Time to event (months)^b^	FU (months)^b^
Biologic grafts	Brunner [20]	R	123	PM (*n* = 103) BD (*n* = 20)	14	20%	3.3	NR	29 (6–58)
	Gleditsch [19]^a^	R	13	PM	100	0%	15.4	3	29
	Albayati [36]	R	51	BD	18	8%	5.9	NR	23 ± 1
	Fu [27]^a^	R	199	BD (*n* = 198) PM (*n* = 1)	49	17%	6.0	37 IQR (27–52)	47 (IQR 29–63)
	Franceschilli [43]	P	100	PM	0	5%	14.0	24–36	20 (6–54)
	Mehmood [42]	RCT	51	BD	100	35%	0	–	12
	Wahed [37]	P	65	PM	42	26%	4.6	14	12 (1–29)
Synthetic grafts	Farag [38]	P	60	PP	100	0%	3.3	NR	12
	Laitakari [44]	RCT	26	PE	20	0%	15.4	NR	60
	Postillon [16]	R	76	PP	100	13%	13.2	NR	31 (2–92)
	Tsunoda [21]	R	50	PP	100	19%	10.0	NR	49 (6–92)
	Hidaka [3]	RCT	38	PP	100	0%	7.9	NR	73 (IQR 65–82)
	Ahmad [22]	P	58	PVDF-PP	36	14%	5.2	NR	35 ± 14
	Chandra [31]	R	25	PP	100	NR	4.0	48	34 (6–82)
	Gleditsch [19]^a^	R	9	PP^d^	100	0%	11.1	3	29
	Emile [45]	RCT	25	PP	100	0%	8.0	NR	18 ± 5
	Fu [27]^a^	R	32	PP	49	17%	18.8	69 (IQR 62–74)	47 (IQR 29–63)
	van Iersel [24]	R	258	PP	19	4%	4.7	NR^c^	24 ± 22
	Luglio [34]	R	20	PP	100	NR	2.4	NR	12
	Silveira [32]	R	71	PE	34	28%	16.9	mean 17	18
	Horisberger [33]	NR	27	PP	0	NR	3.7	NR	22 (2–39)
	Consten [10]	R	919	PP	26	NR	7.4	24 (1–139)	34 (0.4–144)
	Gosselink [39]	R	91	PP	45	0%	4.4	NR	12
	Owais [28]	P	68	PP^d^ (*n* = 31) PE (*n* = 22) PP (*n* = 14)	26	26%	2.9	NR	42 (IQR 26–61)
	Badrek-Am. [29]	P	48	PP	23	19%	12.5	NR^c^	33 (1–186)
	Maggiori [40]	P	33	PP	61	NR	6.1	13 (11–14)	42 ± 7
	Faucheron [26]	P	175	PE	100	NR	1.1	15 (6–24)	74 (24–181)
	Wijffels [30]	P	80	PP	100	41%	2.5	11 (6–16)	23 (2–82)
	Boons [35]	P	65	PP	100	NR	1.5	12	19 (3–41)
	Collinson [41]	P	75	PP	0	NR	5.3	NR	12 (3–48)
	D'Hoore [1]	P	42	PP	100	12%	4.8	73 (54–91)	61 (29–98)

*BD* biodesign, *IQR* inter quartile range, *N* number, *NR* not reported, *P* prospective, *PE* polyester, *PM* permacol, *PP* polypropylene, *PVDF* polyvinylidene fluoride, *R* retrospective, *RCT* randomized controlled trial, *RP* rectal prolapse

^a^Results of studies evaluating a mix of synthetic and biologic grafts are split up

^b^Mean ± SD or median (range) or IQR

^c^Original articles did include Kaplan–Meier analysis

^d^With a component of another synthetic material

The eligible studies reporting on recurrence included 2973 patients, of which 2371 (80%) were treated with a synthetic mesh and 602 (20%) with a biologic graft. Median or mean follow-up ranged from 12 to 74 months for synthetic mesh studies and from 12 to 47 months for biologic mesh studies. Eighteen (62%) studies [1, 3, 10, 16, 19–22, 24, 26–31, 36, 40, 44] had a median or mean follow-up of 2 years or longer.

In total, 155 recurrences were diagnosed in the synthetic mesh group at the end of follow-up, with incidences ranging from 1.1 to 18.8% among studies. Again, polypropylene was the most widely used synthetic implant (*N* = 1978), followed by polyester (*N* = 294).

In the biologic mesh group, 38 patients were diagnosed with a recurrence, with reported proportions ranging from 0 to 15.4% among the included studies. Permacol was used in 282 patients and Biodesign in 320 patients.

**Fig. 3:**
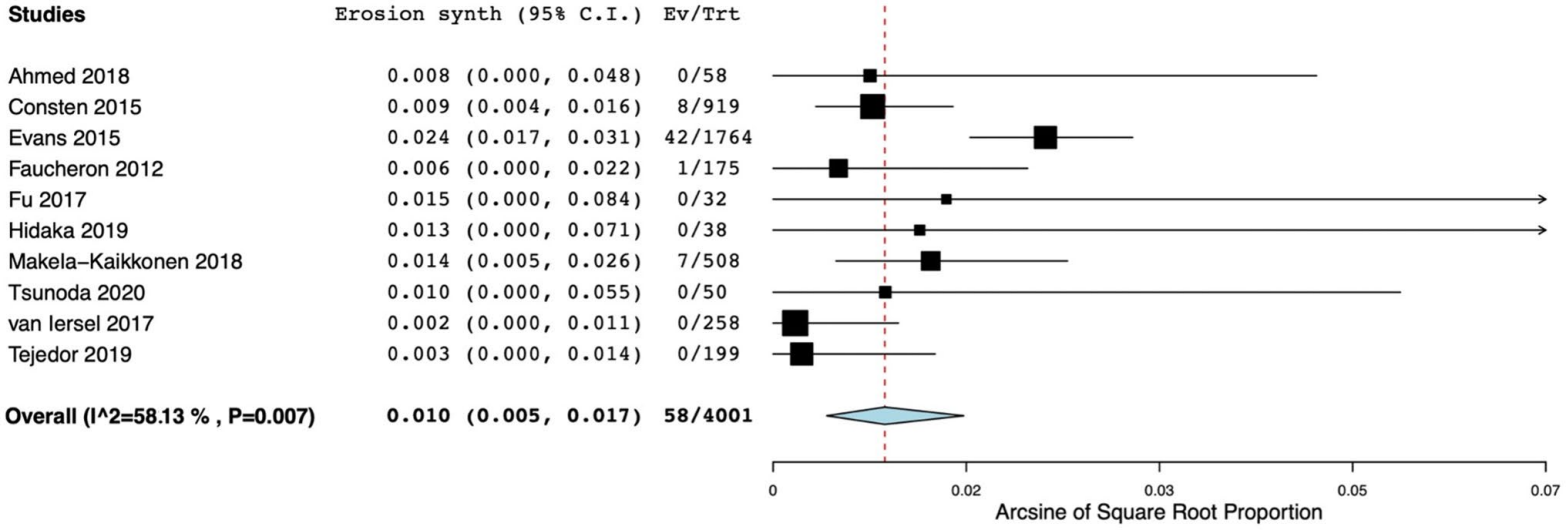
Forest plot of recurrence rates after ventral mesh rectopexy with synthetic mesh. Horizontal axis for Arcsine of Square proportion; Vertical axis for included studies in meta-analyses. *Synth* synthetic, *C.I.* confidence interval, *Ev* events, *Trt* total treated with synthetic mesh

**Fig. 4:**
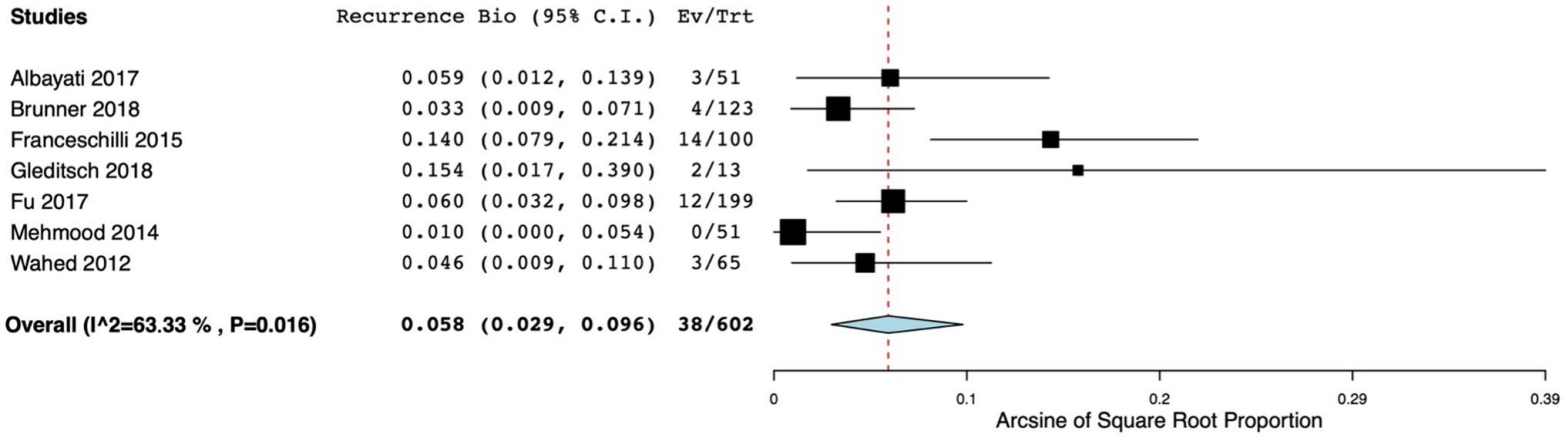
Forest plot of recurrence rates after ventral mesh rectopexy with biologic mesh. Horizontal axis for Arcsine of Square proportion; Vertical axis for included studies in meta-analyses. *Bio* biologic, *C.I.* confidence interval, *Ev* events, *Trt* total treated with biologic mesh

Data could be pooled for both the synthetic and the biologic mesh studies. This resulted in an overall cumulative incidence of recurrence of 6.1% (95% CI 4.3–8.1) and 5.8% (95% CI 2.9–9.6), respectively. Again, there was strong evidence of heterogeneity amongst studies (*I*^2^ = 62% and *I*^2^ = 63%, respectively) (Figs. 3 and 4). Subgroup analyses with (a) studies, including only patients with ERP; (b) studies with an adequate definition of recurrence (defined as two points for item 4 of the MINORS tool); and (c) studies with a prospective design were performed. This was possible only for synthetic mesh studies (the number of the biologic mesh studies was too small to perform subgroup analysis). However, high heterogeneity persisted after conducting the aforementioned subgroup analyses.

## Discussion

Since its introduction, many studies have been published on VMR for rectal prolapse, with promising results regarding functional outcome and recurrence, and with a low incidence of morbidity. However, since the FDA report in 2011 and the subsequent ban in 2019 on synthetic transvaginal mesh, concerns about transabdominal mesh implantation, as in VMR, also appeared. Although transvaginal mesh implantation cannot be compared one to one with transabdominally placed mesh, these concerns stem from a low but undeniable percentage of mesh-related complications seen after VMR. This has led to the development of alternatives such as biologic grafts. Biologic mesh implants are thought to reduce the risk of mesh-related complications attributed to the degradation of the implant over time. However, the process of gradual degradation has led to skepticism amongst some surgeons regarding the risk of recurrence. Previous reviews have not found significant differences in the risk of recurrence or mesh exposure between synthetic versus biologic mesh implants [9, 13]. However, the number of studies reporting on biologic mesh was small, and the follow-up limited. Since the publication of these reviews, new studies on biologic mesh VMR with longer follow-up have been published. With this systematic review, we aimed to compare mesh-related complications and recurrences after synthetic VMR and biologic VMR, based on the best available evidence.

### Mesh-related complications

Of the 34 included studies in this review, eleven studies were considered for analysis of mesh-related complications. Based on the MINORS scores of these studies on mesh-related complications, the overall quality of the included papers was low. All of the three studies that used biologic mesh had a retrospective design, as well as the majority of the ten synthetic mesh studies. Synthetic mesh-related complications ranged between 0 and 2.4%. In performing our meta-analysis, we found a weighted mean of 1.0% (95% CI 0.5–1.7), but with a high heterogeneity. Biologic mesh-related complications ranged between 0 and 0.7%, but data could not be pooled due to the limited number of studies. Based on these data, we can only state that the incidences of mesh-related complications are low for both types of implants. There seems to be a small reduction in mesh-related complications when a biologic mesh is used.

Next to a considerable statistical heterogeneity, there is also a high clinical heterogeneity that make these results even more difficult to interpret. First of all, this holds true for the different types of mesh material used in the synthetic mesh group as well as biologic mesh group, both within and in between studies. In the synthetic mesh group, the two most widely implanted grafts were polypropylene and polyester mesh. Previous studies have suggested that polyester mesh gives a higher risk of mesh exposure compared to polypropylene implants [12]. Although the majority of patients in the synthetic mesh group were treated with a polypropylene implant, still a large group received a polyester mesh. This should be taken into consideration when interpreting the results on synthetic mesh regarding mesh complications.

The two biologic implants encountered in our review were Permacol and Biodesign. The main difference between these two is that Permacol is manufactured of a cross-linked mesh, where Biodesign is made of non-cross-linked mesh. Cross-linking is a chemical process to stabilize collagen fibers and reduce the process of degradation. In animal studies, it has been found that non-cross-linked mesh is rapidly infiltrated with host cells and vessels, whereas cross-linked mesh becomes encapsulated [46]. It can be hypothesized that the fast integration of non-cross-linked material leads to a lower risk of mesh exposure compared to cross-linked mesh. Indeed, all three mesh-related complications in biologic VMR were seen in patients treated with Permacol mesh. However, numbers being so small, it is not justifiable to draw conclusions on the possibly differing risks of mesh complications between Permacol and Biodesign in VMR.

Another difference we found between studies is the type of material used to secure the mesh to the rectal wall. Of the eleven studies included for mesh-related complications, six described the use of non-absorbable sutures [3, 10, 20, 22–24], two authors used absorbable sutures [25, 27], two used endofascial staplers [21, 26] and in one study the type of material was not mentioned [12]. Several study groups have suggested that the material used for securing the mesh to the ventral aspect of the rectum may have an effect on the development of mesh exposure [12, 25]. This concern was also expressed in a consensus statement made by a panel of experts [47], and seems to be coherent with the finding that mesh exposure is frequently found at sites where the mesh was sutured to the rectum and sometimes even seems limited to the exposure of a non-absorbable suture, without direct mesh exposure. In a recent retrospective case-matched study by Tejedor et al. a risk difference in erosion of zero in absorbable sutures versus 3.3% in non-absorbable sutures was found after 6 months of follow-up [25]. Hence, the differences found in applied suture materials could have a confounding influence on the reported incidence of mesh exposure as described in the original studies.

Finally, when interpreting the results of mesh-related complications, one should keep in mind that mesh exposure can begin as an asymptomatic condition. This means that if physical examination is performed on indication only (i.e. in case of complaints), the reported incidences of mesh exposure could be underestimated. Studies that described standard physical examination for all patients at the end of follow-up, were given the maximum score regarding item 4 of the MINORS tool for mesh-related complications. Only the studies of Evans, Faucheron and Fu [12, 26, 27] were given this maximum score and thus it is likely that detection bias effects the results on mesh-related complications. This accounts especially for synthetic mesh studies, where five out of 10 studies had a suboptimal item-4 score and reported zero mesh complications (Table 3). In the three studies with an adequate definition of mesh exposure, both synthetic and biologic grafts were studied with adequate follow-up periods of 3 years or longer.

### Recurrence

The overall quality of studies reporting on recurrence was considered low to medium as assessed with the MINORS tool. Apart from those, five studies were scored as high quality.

Altogether, 29 studies reported on the risk of recurrence after VMR, of which a majority of 22 studies used synthetic mesh only. Studies using biologic grafts consisted mainly of retrospective cohorts, whereas in synthetic mesh studies data were collected prospectively in half of the studies. The incidence of recurrence ranged from 0 to 15.4% after biologic VMR and from 0 to 18.8% after synthetic VMR. Pooling of the data resulted in a weighted mean of 5.8% (95% CI 2.9–9.6; *I*^2^ = 63%) and 6.1% (95% CI 4.3–8.1; *I*^2^ = 62%) for biologic and synthetic VMR, respectively. Based on these data, there appears to be a comparable risk on recurrence. Again, the substantial statistical and clinical heterogeneity makes it difficult to draw definitive conclusions.

An important factor influencing the clinical heterogeneity here is the indication for surgery. There were cohorts with ERP patients only, those with IRP patients only, and mixed cohorts.

Although (recurrence of) ERP is a rather clear diagnosis that can be confirmed on physical examination, a (recurrent) symptomatic IRP is less straight forward. This disparity is due to the fact that an IRP is not always accompanied by functional complaints [48, 49]. Therefore, when anatomical abnormalities are found (best seen on imaging studies rather than on physical examination) it is mandatory to ask for accompanying functional symptoms. This difficulty was reflected in the various definitions of recurrence and follow-up routines in the included studies (ranging from routinely performed postoperative imaging and/or physical examination to clinical examination only in case of complaints). We deemed a recurrent prolapse adequately diagnosed only when an anatomical recurrence on physical examination and/or on imaging studies (defecogram or dynamic MRI) was linked to functional complaints and evaluated on a routine basis. If studies used a similar definition, they were given the maximum score regarding item 4 for recurrence with the MINORS tool. Only thirteen studies [3, 16, 19, 21, 26, 27, 34, 35, 38, 39, 42, 43, 45] were given the maximum score for item 4. Once again, this might have led to detection bias.

Another confounding factor was the lack of homogeneity amongst studies regarding the number of patients that had undergone a previous surgical intervention for rectal prolapse, with proportions ranging from 0 to 41% (Table 4).

Subgroup analysis for recurrence was performed with those studies analyzing only ERP patients, those with an adequate definition of recurrence and finally those with prospective study design. Unfortunately, statistical heterogeneity remained high.

For both outcome measures of interest, the chance of an event occurring depends on the duration of follow-up. Unfortunately, with the available data, it was not possible to make a disease-free survival analysis with time-to-event as an additional test variable. Although the majority of studies had a mean or median follow-up of 2 years or longer, standard deviations were often large and ranges were wide. Only two studies [3, 26] reporting on mesh-related complications and 4 studies [1, 3, 26, 44] presenting data on recurrence had a mean or median follow-up longer than 5 years. These were all synthetic mesh cohorts. We suggest future research to focus on long-term follow-up (more than 5 years).

A strong point of this systematic review is that we have done the utmost to limit selection bias by contacting the corresponding authors of papers where additional data was needed to make them eligible for this review. This led to seven extra papers [16, 19–21, 23, 33, 34] deemed suitable for inclusion. Nonetheless, 10 papers [11, 50–58] still had to be excluded due to non-response.

## Conclusions

In conclusion, the overall low to medium quality of the available studies and lack of homogeneity make it difficult to draw definitive conclusions on the topic of choice of mesh implant in VMR regarding mesh exposure and recurrence. However, based on the available literature and the pooled data analysis in this systematic review, we found no evidence to support the idea that biologic mesh entails a higher risk of recurrence compared to synthetic mesh in VMR after medium-term follow-up. There might be a small advantage of a lower risk on mesh-related complaints in favor of biologic mesh. This possible advantage has to be considered against the higher costs of biologic mesh.

## Data Availability

Available upon request.
